# Cyanobacterial Variability in Lichen Cephalodia

**DOI:** 10.3390/jof9080826

**Published:** 2023-08-05

**Authors:** Maria Prieto, Natalia Montané, Gregorio Aragón, Isabel Martínez, Clara Rodríguez-Arribas

**Affiliations:** Biodiversity and Conservation Area, Department of Biology and Geology, Physics and Inorganic Chemistry, Rey Juan Carlos University, C/Tulipán s/n, Móstoles, 28933 Madrid, Spain; nmontane3@gmail.com (N.M.); gregorio.aragon@urjc.es (G.A.); isabel.martinez@urjc.es (I.M.); clara.rodriguez@urjc.es (C.R.-A.)

**Keywords:** cyanolichens, intrathalline, rbcLX, *Nephroma antarcticum*, *Nostoc*, *Pannaria farinosa*, *Pseudocyphellaria granulata*, specialization, symbiosis

## Abstract

The ecological success of lichens is related to both myco- and photobionts which condition the physiological limits of the lichen symbioses and thus affect their ecological niches and geographic ranges. A particular type of lichen, called cephalolichen, is characterized by housing both green algal and cyanobacterial symbionts—the latter is restricted to special structures called cephalodia. In this type of lichen, questions related to specialization within species or within individuals are still unsolved as different patterns have previously been observed. In order to study the variability at the intrathalline, intraspecific, and interspecific level, cyanobionts from different cephalodia within the same thalli and from different thalli were genetically analysed in three cephalolichen species at two different forests (18 thalli, 90 cephalodia). The results showed variability in the cephalodial *Nostoc* OTUs in all the studied species, both at the intrathalline and intraspecific levels. The variability of *Nostoc* OTUs found in different cephalodia of the same thallus suggests low specialization in this relationship. Additionally, differences in OTU diversity in the three studied species and in the two forests were found. The variability observed may confer an increased ecological plasticity and an advantage to colonize or persist under additional or novel habitats or conditions.

## 1. Introduction

Lichenization is a successful nutritional strategy, with almost 20% of all fungal species being lichenized [1] and dominating about 8% of the land surface of the world [2]. According to photobiont association, lichens are divided into two groups. Bipartite lichens are characterized by a mycobiont establishing with green algae (chlorolichen) or cyanobacteria (cyanolichen), in which the photosynthetic partner (phycobiont) provides carbon products to the mycobiont. In tripartite lichens (cephalolichens), the fungus is simultaneously associated with both green algae (photobiont) and cyanobacteria (cyanobiont). In these lichens, the photobiont is distributed through the thallus, providing fixed carbon, and the cyanobiont is often confined to special structures, called cephalodia, supplying the fixed nitrogen. In this case, cyanobionts are specialized for nitrogen fixation; they are predominantly heterotrophic and show an increase in heterocyst proportions over 30% of non-symbiotic cyanobacterial cells and lichenized cyanobionts of bipartite cyanolichens [3,4].

Although lichens are compound organisms, and the physiological limits of the lichen symbiosis are driven by the association as an integrated whole, some aspects are specific to the photosynthetic symbiont [5]. Thus, photobionts are involved in photosynthesis, secondary compound production, nitrogen fixation, etc., and their preferences regarding abiotic conditions may limit the ecological niches and geographic ranges of lichens [6,7,8,9]. In this respect, the specialization of the fungal–photobiont association is related to the potential acclimation to local environmental conditions and colonization of new niches and geographic regions. Thus, low specialization between myco- and photobionts may preclude limitations if mycobionts have the ability to lichenize with different photobionts [10] as they can expand their ecological niches and geographic distributions outside the physiological limits imposed by a single photobiont species. On the other hand, specialized species may have narrower geographical distributions and ecological niches [11,12]. While there are different attributes to characterize specialization in biotic interactions, the classification of organisms as specialists or generalists has been mostly based on the number of interacting partners [13,14]; however, see [15]. The variability in the photobiont partner is, thus, related to the specialization of the mycobiont for the photobiont and may be observed at different levels (biological, geographical, or ecological) [10,16,17,18,19,20]. At the biological level (individual, species, or genus levels), numerous studies have shown contrasting patterns: from a high specialization of mycobiont towards the photobiont, i.e., [11,21,22,23,24], to a common pattern of generalization (i.e., a high number of interacting photobionts) [25,26,27]. When comparing specialization between bi-membered (cyanolichens and chlorolichens) and tri-membered (cephalolichens) lichen species, several patterns have been found. A high specialization hypothesized for cephalolichens [28] differed from the results of [20], showing that cephalolichens are more generalized than cyanolichens, and from other studies, which found no differences between both [25,29]. At the intrathalline (individual) level, and concerning cephalolichens specifically, it has been shown that most tripartite lichens contain the same *Nostoc* strain in all cephalodia of individual thalli [28], with the exception of *Peltigera venosa*, *Lobaria pulmonaria*, and three species of *Pannaria* (*P. farinosa*, *P. sphinctrina*, and *P. lobulifera*), which housed different cyanobionts in different cephalodia [23,30,31]. However, this conclusion is based on very few studies analysing specifically the intrathalline variation in cephalodia. Additionally, concerning species with green algae, it has been shown that the co-occurrence of several photobionts in individual lichen thalli is relatively common [10,32,33,34,35]. At the community scale, it has been revealed that lichens do not show a one-to-one relationship as different species of cyanobacteria are shared between different lichen species [21,31,35,36], like the Lichen Guilds theory proposes [37,38].

On the other hand, factors driving photobiont selection are diverse and related to phylogenetic relationships, reproductive strategy, the availability of photobionts, or ecological factors [12,18,24,29,36,39,40,41,42]. In a recent study, the authors showed that photobiont availability, the function of the cyanobiont (principal photobiont for cyanolichens or secondary photobiont for cephalolichens), and the mycobiont species were factors related with specialization in cyanobacterial lichens [20].

Based on this previous study, of which shows a high variability of *Nostoc* phylogroups in different lichen species along a latitudinal gradient in Chile, a question arose on whether this variability was also present at the intrathalline (individual) level, and if this variation may be related with the geographic gradient studied. As stated before, this variability had been observed in several tripartite lichen species previously [23,30,31]. Thus, the aims of this study were to investigate if the individual thalli of tripartite lichens host different *Nostoc* genotypes depending on the species and on the studied forest. For this purpose, three cephalolichens species were selected at two different forests at different latitudes in Chile, and different cephalodia per thallus were genetically characterized.

## 2. Materials and Methods

Two forests from a previous study carried out in Chile and sampled between 2017 and 2018 were used (Parque Nacional Torres del Paine and Isla Navarino) [20]. Forest stands were mostly formed by *Notofagus pumilio* with over 65% of cover. Within these forests, three species of Peltigerales with cephalodia were collected: *Pannaria farinosa*, *Nephroma antarcticum*, and *Pseudocyphellaria granulata*. From each species, 6 thalli were selected (3 at each forest), and from each thallus, 5 different cephalodia were analysed. Samples were air-dried and stored at −20 °C. DNA from different cephalodia in each thallus was extracted using Chelex^®^ 100 Chelating Resin (Bio-Rad, Hercules, CA, USA). Region rbcLX was amplified using primers CW and CX [43] and the following program: 95 °C 15 min; 35 cycles of 1 min at 95 °C, 30 s at 54 °C, 30 s at 72 °C; and 10 min at 72 °C. The PCR products were sequenced at Macrogen Spain service (www.macrogen.com (accessed on 21 September 2020)) using the same primers employed in the PCR.

The obtained sequences were edited and aligned using Geneious Prime v. 2021.0.1 software (https://www.geneious.com (accessed on 11 January 2021)). Some samples failed in the PCR or were too short and were discarded. Ambiguous regions and introns were delimited manually and excluded for the analysis using AliView v. 1.26, Uppsala, Sweeden [44]. *Nostoc* sequences were grouped into operational taxonomic units (OTUs). The delimitation of Nostoc OTUs was based on the ASAP method (Assemble Species by Automatic Partitioning) [45], which proposes partitions of species hypotheses using genetic distances calculated between DNA sequences. Additionally, we performed a phylogenetic and network analysis. The ASAP analysis was carried out in the webserver (https://bioinfo.mnhn.fr/abi/public/asap/ (accessed on 9 March 2021)), applying the Jukes–Cantor (JC69) model of substitution (groups below 0.01 probability were split, the 10 best scores were kept, and −1 was the seed value). Partitions included in the 0.001–0.01 range of genetic distances were selected. A maximum likelihood phylogenetic analysis (ML) was conducted with RAxML v. 8.2.12, Karlsruhe, Germany [46], assuming the GTRGAMMA model. The node support was estimated with the rapid bootstrap algorithm, using 1000 pseudoreplicates. A haplotype network was constructed using the TCS method [47], as implemented in PopART v. 1.7, Dunedin, New Zeland (http://popart.otago.ac.nz (accessed on 17 July 2023)).

## 3. Results

**Figure 1:**
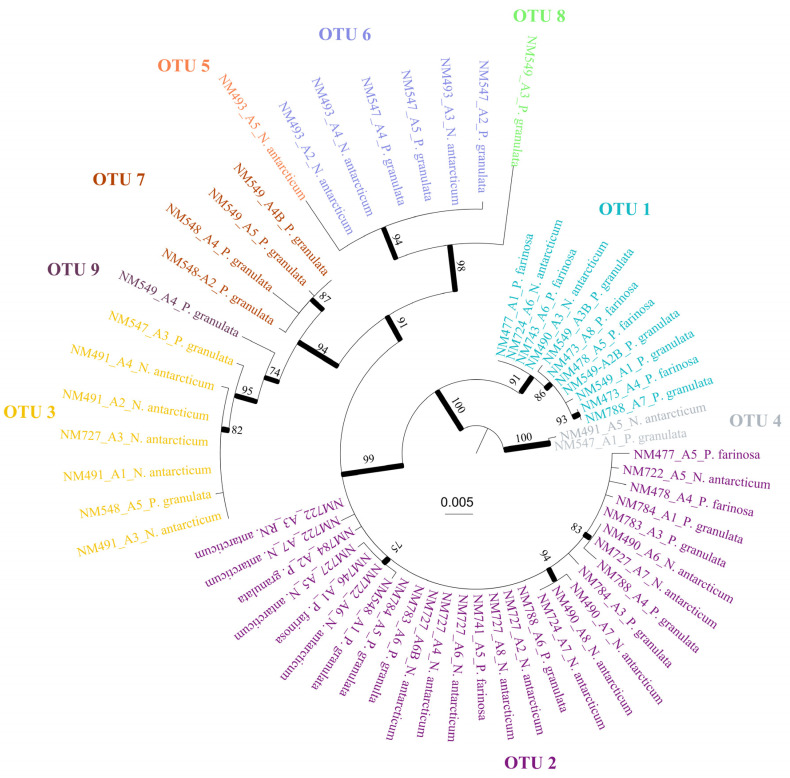
Best tree from the ML analysis of the rbcLX region. Bootstrap values ≥70% are indicated on or below the branches and with thicker lines. OTUs delimited based on the ASAP results are depicted in the tree and represented with different colours.

**Figure 2:**
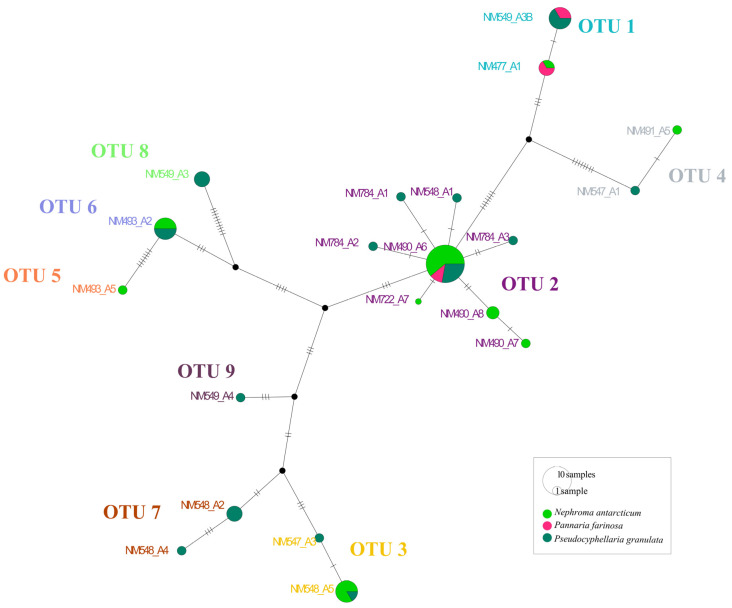
The TCS haplotype network for *Nostoc* rbcLX sequences. The size of the pie chart is proportional to the number of thalli belonging to the haplotype, and the colour is according to the 3 studied species. The dash on the line represents one mutational step of the haplotype sequence. Black-filled circles indicate missing haplotypes. Haplotypes are grouped in OTUs obtained by the ASAP analysis.

**Figure 3:**
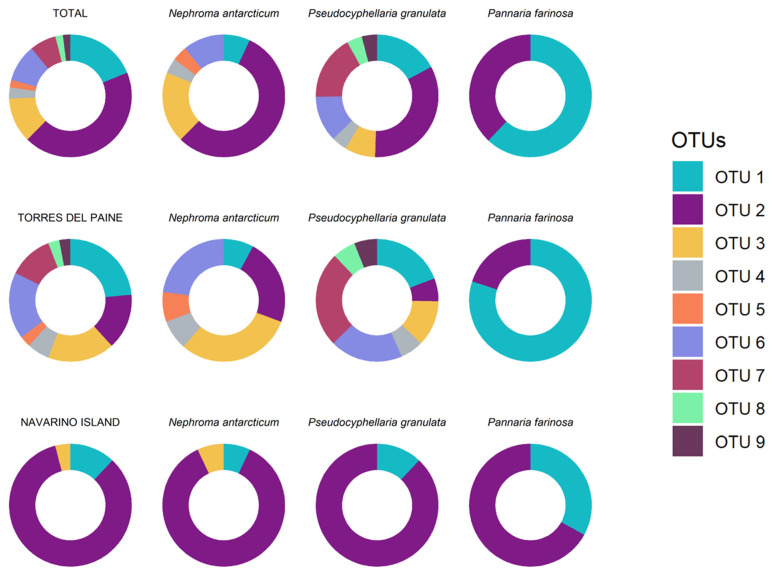
The abundance (richness) of *Nostoc* OTUs found in the studied species at the different levels.

A total of 61 cyanobacterial consensus sequences were obtained (Table 1). The best partition obtained in ASAP was selected based on the lower score. Thus, a total of nine OTUs were found consorting with the three species (Table 1). This result is congruent with the ML phylogenetic analyses and the haplotype network, as shown in Figure 1 and Figure 2. *Nostoc* OTUs were shared between the species. Thus, OTUs 2 (present in 25 cephalodia out of 58), 1 (11), 3 (7) and 6 (6) were the most abundant, while the rest of OTUs (except 4) were only found in 1 cephalodia (Table 1; Figure 3).

The obtained results were different in the different species and in both forests (Table 1 and Figure 3). Thus, *Pseudocyphellaria granulata* had the highest variability in OTUs followed by *Nephroma antarcticum*, which also showed high variability. The results in *Pannaria farinosa* showed lower variability in its cyanobionts. In addition, the two OTUs found in *Pannaria farinosa* (OTU 1 and 2) were shared with the other two lichen species. Also, OTUs 3, 4 and 6 were found in both *Pseudocyphellaria granulata* and *Nephroma antarcticum*. On the other hand, OTU 5 was exclusive from *Nephroma antarcticum*, while OTUs 7, 8, and 9 were only found in *Pseudocyphellaria granulata*.

When comparing forests, a significant difference was observed between Torres del Paine and Navarino Island, as the latter showed lower diversity in the cyanobionts found, and there were also differences in their abundances. Nine OTUs were found in Torres del Paine with a dominance of OTU 1; meanwhile, three OTUs were found in Navarino, with OTU 2 being dominant. Nonetheless, the OTUs from Navarino Island were a subset of those from Torres del Paine, showing a nested pattern.

The three species also showed differences in their cyanobionts depending on the forest. *Nephroma antarcticum* had a similar proportion of OTUs 2 and 3 in Torres del Paine, while *Pseudocyphellaria granulata* had a higher proportion of OTU 7. All three species showed a dominance of OTU 2 in Navarino.

All the species showed variability in their cephalodial *Nostoc* at the intrathaline (individual) and intraspecific levels. All thalli except one of *N. antarcticum* (five in total) had two different OTUs per thallus. In *Pseudocyphellaria granulata*, three thalli presented between three and four *Nostoc* OTUs. In this species, two thalli from Navarino only had one OTU and other sample had two OTUs. Despite the poor results obtained in *Pannaria farinosa*, as only nine cephalodia were successfully sequenced, the studied thalli had two OTUs.

## 4. Discussion

In relation to the variability of the photobiont partners in lichenized fungi, many basic questions remain unknown. An important step is to learn about this variability of photobionts in lichens at different levels, from the individual to the ecological communities. The results from this study, based on the richness of cyanobionts in tripartite lichens (cephalolichens), showed differences in the *Nostoc* OTUs from cephalodia at different scales: thallus, species, and forests.

At the lowest level, the thallus (individual), a high variability of cyanobionts was found in different cephalodia of the same thallus. Although there was a difference in the number of OTUs per thalli between the three studied species, all three showed the ability to harbour more than one and different *Nostoc* OTUs within a thallus. The coexistence inside a single lichen thallus of different *Trebouxia* species has been previously demonstrated [26,48,49,50], but it has rarely been studied in tripartite lichens (e.g., in *Lobaria pulmonaria*, *Peltigera venosa* and *Pannaria*) [23,30,31]. In the case of *Lobaria pulmonaria* [23], only one of the studied thalli showed different *Nostoc* genotypes in different cephalodia. The results obtained in *Pannaria* showed non identical *Nostoc* 16S sequences from different cephalodia in the same thallus in three *Pannaria* individuals belonging to *P. farinosa*, *P. sphinctrina* and *P. lobulifera* [30]. However, the genetic distance between these sequences is not enough to consider them as different haplotypes, at least in *P. sphinctrina* and *P. lobulifera*. In addition, previous morphological studies not using molecular data observed different cyanobacterial morphotypes within single thalli and occasionally even in the same cephalodium [51,52].

The ecological significance of photobiont coexistence in the same thallus is not clear. Additionally, it is not clear if this occurrence is a widespread phenomenon. On the one hand, the same mycobiont with different photobionts has shown differences in various aspects of its physiology [53]. On the other hand, [50] demonstrated that the contribution of the secondary photobionts was marginal in most thalli. Additionally, [31] suggested the possibility of the existence of different degrees of lichenization with different *Nostoc* strains, ranging from loosely associated colonies to well-corticated cephalodia in *Peltigera venosa*. It is unlikely that a loose association between the myco- and cyanobiont occurs in cephalodia—it has been shown that there is a high specific biorecognition process involved in the acquisition of the cephalodial *Nostoc* [54], performed by specific lectins produced and secreted by the mycobiont. Thus, lectins produced by a vegetative fungal component in *Peltigera aphthosa* were shown to have a similar function in selecting the compatible cephalodial cyanobacterium as lectins produced by germinating spores. The observed photobiont diversity was already predicted to operate on the level of single cyanolichen thalli—especially in the case of cephalodiate species [55].

At the species level (comparing different thalli from the same species), numerous studies have shown contrasting patterns: from a high specialization of mycobiont towards the photobiont [11,21,22,23,24], to a common pattern of generalization (i.e., a high number of interacting photobionts) [25,26,27,30]. Previous results [21] have shown that the identity of cyanobionts was related with the species identity of the lichen-forming fungus rather than the geographical area where the lichen was growing. In this previous example, the same lichen species collected in Sweden and Finland (i.e., *Peltigera aphthosa*, *P. canina* and *Nephroma arcticum*) had the same identical intron sequences in different samples of the same lichen species. Conversely, in the present study, the location (forests) determined the cyanobiont variability. Thus, in Navarino Island, there was a lower diversity of cyanobionts for the three studied species, suggesting that environmental variables may determine the cyanobiont pool found in ecological communities [20].

In many cases [22,56], one fungal species can associate with more than one symbiont, and these are often also shared by several taxonomically unrelated cyanolichen species. This is also in line with the Lichen Guild theory [36], where different species shared the same *Nostoc* genotype. As in our results, the most common *Nostoc* OTUs were shared between the three species, pointing out the existence of facilitation in a community context where some species may act as core species (source of cyanobionts), whereas others may act as fringe species, capturing their photobionts from the former ones [57]. Species with the ability to associate with many OTUs (generalized) may host compatible OTUs for other more specialized species, with the former acting as a core species, as could be *Pseudocyphellaria granulata* in the current study.

Several mechanisms have been proposed to explain the differences in specialization in lichens. Previous studies suggest that geographic and ecological factors including macroclimatic variables can drive the differences in the specialization of the association in both cyano- and chlorolichens [10,42]. In general, a low specialization towards phycobionts allows for the host to associate with ecologically diversified or locally adapted algae, thereby broadening the lichen ecological amplitude [6,34]. For instance, a previous study analysed *Nostoc* cyanobionts from five lichen species in maritime Antarctica and determined that lichens from those regions were more generalized than lichens from temperate and boreal regions; this was regarded as lichens adapting to extreme environmental stress [25]. However, this finding opposed the results obtained here. In addition, environmental factors have been found to affect the *Nostoc* pool at the community scale [20]. In Navarino Island, the diversity of *Nostoc* genotypes found was lower than in Torres del Paine, harbouring a subset of the genotypes found at northern locations (i.e., Torres del Paine). The ability of mycobiont species to interact with different cyanobionts in different localities, even though they are present, may allow for some fungal hosts to associate with the cyanobacterial genotypes that are optimally adapted to the conditions [55].

Our study also notices the high variability of *Nostoc* OTUs (low specialization) at different biological and spatial scales in cephalolichens. This high variability of partners, even at the intrathalline level, could be due to a low dependence on the cyanobiont in those tri-membered lichens, as the main photosynthetic partner is green algae, which conducts the photosynthetic activity, whereas the cyanobiont’s main function is nitrogen fixation [28,58]. In addition, this study emphasizes the importance of the contextualization of the scale, as results based on one site could change when widening the spatial scale, and thus limit the understanding of specialization. Also, it highlights the importance of determining the local availability of cyanobionts, as differences in specialization between locations may be due to a different pool of the cyanobionts available to interact.

## Figures and Tables

**Table 1:** Voucher and GenBank numbers of the species studied with thalli, cephalodia, and forest information and OTU classification based on the ASAP analysis, ML phylogenetic analysis, and TCS haplotype network. TP: Torres del Paine, IN: Navarino Island.

Species	Thallus	Cephalodia	OTU ASAP	Forest	Voucher	GenBank
*Nephroma antarcticum*	NM490	A3	1	TP	ARAN20283	OR344442
*Nephroma antarcticum*	NM490	A6	2	TP		OR344444
*Nephroma antarcticum*	NM490	A7	2	TP		OR344445
*Nephroma antarcticum*	NM490	A8	2	TP		OR344446
*Nephroma antarcticum*	NM491	A3	3	TP	ARAN20284	OR344447
*Nephroma antarcticum*	NM491	A1	3	TP		OR344449
*Nephroma antarcticum*	NM491	A2	3	TP		OR344450
*Nephroma antarcticum*	NM491	A4	3	TP		OR344451
*Nephroma antarcticum*	NM491	A5	4	TP		OR344448
*Nephroma antarcticum*	NM493	A5	5	TP	ARAN20285	OR344452
*Nephroma antarcticum*	NM493	A2	6	TP		OR344454
*Nephroma antarcticum*	NM493	A3	6	TP		OR344455
*Nephroma antarcticum*	NM493	A4	6	TP		OR344456
*Nephroma antarcticum*	NM722	A3	2	IN	ARAN20292	OR344474
*Nephroma antarcticum*	NM722	A6	2	IN		OR344476
*Nephroma antarcticum*	NM722	A5	2	IN		OR344475
*Nephroma antarcticum*	NM722	A7	2	IN		OR344477
*Nephroma antarcticum*	NM724	A6	1	IN	ARAN20293	OR344478
*Nephroma antarcticum*	NM724	A7	2	IN		OR344479
*Nephroma antarcticum*	NM727	A3	3	IN	ARAN20294	OR344481
*Nephroma antarcticum*	NM727	A2	2	IN		OR344480
*Nephroma antarcticum*	NM727	A4	2	IN		OR344482
*Nephroma antarcticum*	NM727	A6	2	IN		OR344484
*Nephroma antarcticum*	NM727	A8	2	IN		OR344486
*Nephroma antarcticum*	NM727	A5	2	IN		OR344483
*Nephroma antarcticum*	NM727	A7	2	IN		OR344485
*Pannaria farinosa*	NM473	A4	1	TP		OR344431
*Pannaria farinosa*	NM473	A8	1	TP	ARAN20286	OR344432
*Pannaria farinosa*	NM477	A1	1	TP	ARAN20287	OR344433
*Pannaria farinosa*	NM477	A2	2	TP		OR344434
*Pannaria farinosa*	NM478	A5	1	TP	ARAN20288	OR344441
*Pannaria farinosa*	NM741	A5	2	IN	ARAN20295	OR344487
*Pannaria farinosa*	NM743	A6	1	IN	ARAN20296	OR344488
*Pannaria farinosa*	NM746	A1	2	IN	ARAN20297	OR344489
*Pseudocyphellaria granulata*	NM547	A4	6	TP	ARAN20289	OR344460
*Pseudocyphellaria granulata*	NM547	A5	6	TP		OR344461
*Pseudocyphellaria granulata*	NM547	A2	6	TP		OR344458
*Pseudocyphellaria granulata*	NM547	A3	3	TP		OR344459
*Pseudocyphellaria granulata*	NM547	A1	4	TP		OR344457
*Pseudocyphellaria granulata*	NM548	A5	3	TP	ARAN20290	OR344465
*Pseudocyphellaria granulata*	NM548	A2	7	TP		OR344462
*Pseudocyphellaria granulata*	NM548	A4	7	TP		OR344463
*Pseudocyphellaria granulata*	NM548	A1	2	TP		OR344464
*Pseudocyphellaria granulata*	NM549	A4B	7	TP	ARAN20291	OR344469
*Pseudocyphellaria granulata*	NM549	A5	7	TP		OR344470
*Pseudocyphellaria granulata*	NM549	A3	8	TP		OR344471
*Pseudocyphellaria granulata*	NM549	A4	9	TP		OR344472
*Pseudocyphellaria granulata*	NM549	A2B	1	TP		OR344467
*Pseudocyphellaria granulata*	NM549	A3B	1	TP		OR344468
*Pseudocyphellaria granulata*	NM549	A1	1	TP		OR344466
*Pseudocyphellaria granulata*	NM783	A3	2	IN	ARAN20298	OR344491
*Pseudocyphellaria granulata*	NM783	A6	2	IN		OR344492
*Pseudocyphellaria granulata*	NM784	A5	2	IN	ARAN20299	OR344496
*Pseudocyphellaria granulata*	NM784	A2	2	IN		OR344494
*Pseudocyphellaria granulata*	NM784	A1	2	IN		OR344493
*Pseudocyphellaria granulata*	NM784	A3	2	IN		OR344495
*Pseudocyphellaria granulata*	NM788	A7	1	IN	ARAN20300	OR344500
*Pseudocyphellaria granulata*	NM788	A6	2	IN		OR344499

## Data Availability

Sequences produced in this study are deposited in GenBank. Lichen samples used are deposited in Herbarium ARAN-Fungi.
